# High-Dimensional Phospho-CyTOF Characterization of T-Cell Activation Responses in Whole Blood

**DOI:** 10.21769/BioProtoc.5473

**Published:** 2025-10-20

**Authors:** Ilyssa E. Ramos, Teresa S. Hawley, Kevin Rose, Brynja Matthiasardottir, Rohit Farmer, Kyu Lee Han, Michal Toborek, Iyadh Douagi, Georgette N. Jones, James M. Cherry

**Affiliations:** 1 Protein and Chemistry Section (PCS), Research Technologies Branch (RTB), National Institute of Allergy and Infectious Diseases, Bethesda, MD, USA; 2 Center for Human Immunology, Autoimmunity, and Inflammation (CHI), National Institute of Allergy and Infectious Diseases, Bethesda, MD, USA; 3 Department of Biochemistry and Molecular Biology, University of Miami Miller School of Medicine, Coral Gables, FL, USA; 4 Flow Cytometry Section, Research Technologies Branch (RTB), National Institute of Allergy and Infectious Diseases, Bethesda, MD, USA; 5 National Human Genome Research Institute (NHGRI), Bethesda, MD, USA; 6 Computational Biology, Bioinformatics, and Genomics, Biological Sciences, University of Maryland, Baltimore, MD, USA

**Keywords:** Helios CyTOF, Phospho-flow, T-cell stimulation, Stanford Human Immune Monitoring Core (HIMC), StemCell activator cocktail, Whole blood, Cryopreservation, Prot1/Thaw-Lyse

## Abstract

Recent advances in single-cell technologies have provided limited insight into the role of protein phosphorylation in T-cell fate and function. Dysregulated protein phosphorylation is associated with adverse clinical outcomes, emphasizing the need for reliable methods to unravel the complexities of T-cell signal transduction and disease-related alterations. While flow cytometry is widely used, it is constrained by spectral overlap, limiting the number of protein targets for simultaneous analysis. To overcome this, we present a robust protocol for whole blood T-cell stimulation and subsequent analysis using mass cytometry by time-of-flight (CyTOF). CyTOF minimizes spillover into adjacent channels by employing highly pure, stable, heavy metal–conjugated antibodies for protein detection. This protocol offers a high-dimensional approach for phenotypic and phospho-protein characterization of key signaling pathways, including JAK/STAT, MAPK, PI3K/mTOR, PKC, and NF-κB. A key feature is the T-cell stimulation reagent, which mimics endogenous activation by engaging the T-cell receptor (TCR)/CD3 complex and providing co-stimulation via an anti-CD28 antibody. Further, we enhance reproducibility and enable batch processing through the implementation of the Prot1/Thaw-Lyse system for immediate cryopreservation of stimulated blood samples. By employing CyTOF, this method permits the simultaneous analysis of 31 protein targets with single-cell resolution, minimizing spillover and providing superior specificity, sensitivity, and resolution over flow cytometric methods. This approach facilitates the robust assessment of TCR activation and its effect on bystander populations, which has been challenging with spectral flow cytometry due to the limited availability of methanol-resistant fluorophores. This protocol is a precise and reproducible method for elucidating the downstream effects of T-cell stimulation and immune status, with significant potential for clinical applications, including the assessment of T-cell-targeted therapies.

Key features

• Mass cytometry (CyTOF) analysis: A "vein-to-tube" protocol for high-dimensional profiling of key signaling pathways (JAK/STAT, MAPK, PI3K/mTOR, PKC, NF-κB) in human whole blood.

• Cryopreservation for batch processing: Enables long-term storage and standardized batch processing of whole blood samples by implementing immediate cryopreservation via the Prot1/Thaw-Lyse system.

• Assessment of early T-cell activation: This workflow involves a rapid, 15-min stimulation length using a proprietary T-cell activator that provides TCR engagement and co-stimulation.

• Expertise required: Intended for users experienced in surface and intracellular antibody staining techniques for cytometric applications.

## Graphical overview


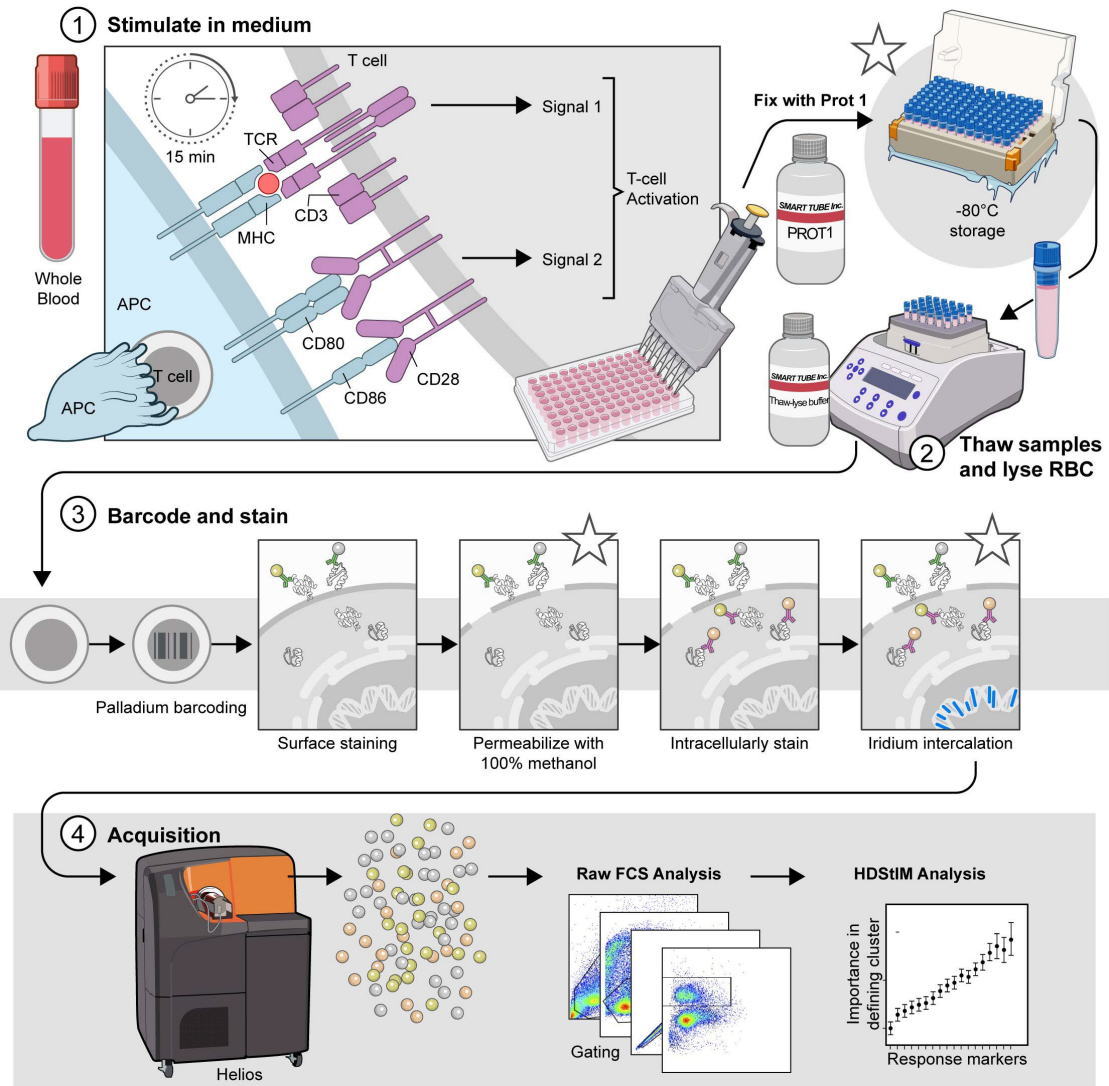


**The comprehensive T-cell Phospho-CyTOF pipeline.** This schematic illustrates the established pipeline for high-dimensional analysis of T-cell phosphorylation using mass cytometry, outlining the critical steps from sample preparation to data acquisition. 1) Stimulate in complete RPMI (cRPMI). Whole blood is stimulated to induce T-cell activation and subsequent phosphorylation events. After 15 min of stimulation, the cells are fixed with Prot1 to preserve cellular signaling states. (*) The fixed samples can be stored at -80 °C for later processing. Stimulated and fixed samples may be stored at -80 °C for approximately 6 months to one year prior to processing. 2) Thaw samples and lyse red blood cells (RBCs). When ready for downstream analysis, fixed samples are thawed and treated with Thaw-Lyse reagent to remove red blood cells. 3) Barcode and stain. Live cells are palladium-barcoded following the manufacturer's specifications to permit multiplexed analysis. Subsequently, cells undergo surface staining with metal-conjugated antibodies. Following surface staining, cells are permeabilized with 100% methanol on wet ice to allow access to intracellular antibodies. (*) The samples can be stored at -80 °C for 4–6 weeks in 100% methanol prior to intracellular staining. When ready, the samples are rehydrated and intracellularly stained with additional metal-conjugated antibodies targeting phosphorylated proteins and intracellular markers. (*) Finally, the cells are incubated overnight at 4 °C with an iridium intercalation solution for DNA staining, which facilitates live/dead cell discrimination and doublet exclusion during acquisition. Avoid storing samples in iridium intercalation solution for more than 12 h before acquisition. 4) Acquire. Before acquisition, samples are resuspended in cell acquisition solution plus (CAS^+^) containing a 1:10 dilution of EQ4 beads and adjusted to a concentration that yields approximately 350 events per second (±50 events per second). Data are acquired using a Helios mass cytometer and transferred for subsequent analysis. Asterisks (*) indicate potential stopping points within the protocol, where samples can be safely stored before proceeding to the next step.

## Background

**Figure 1.**
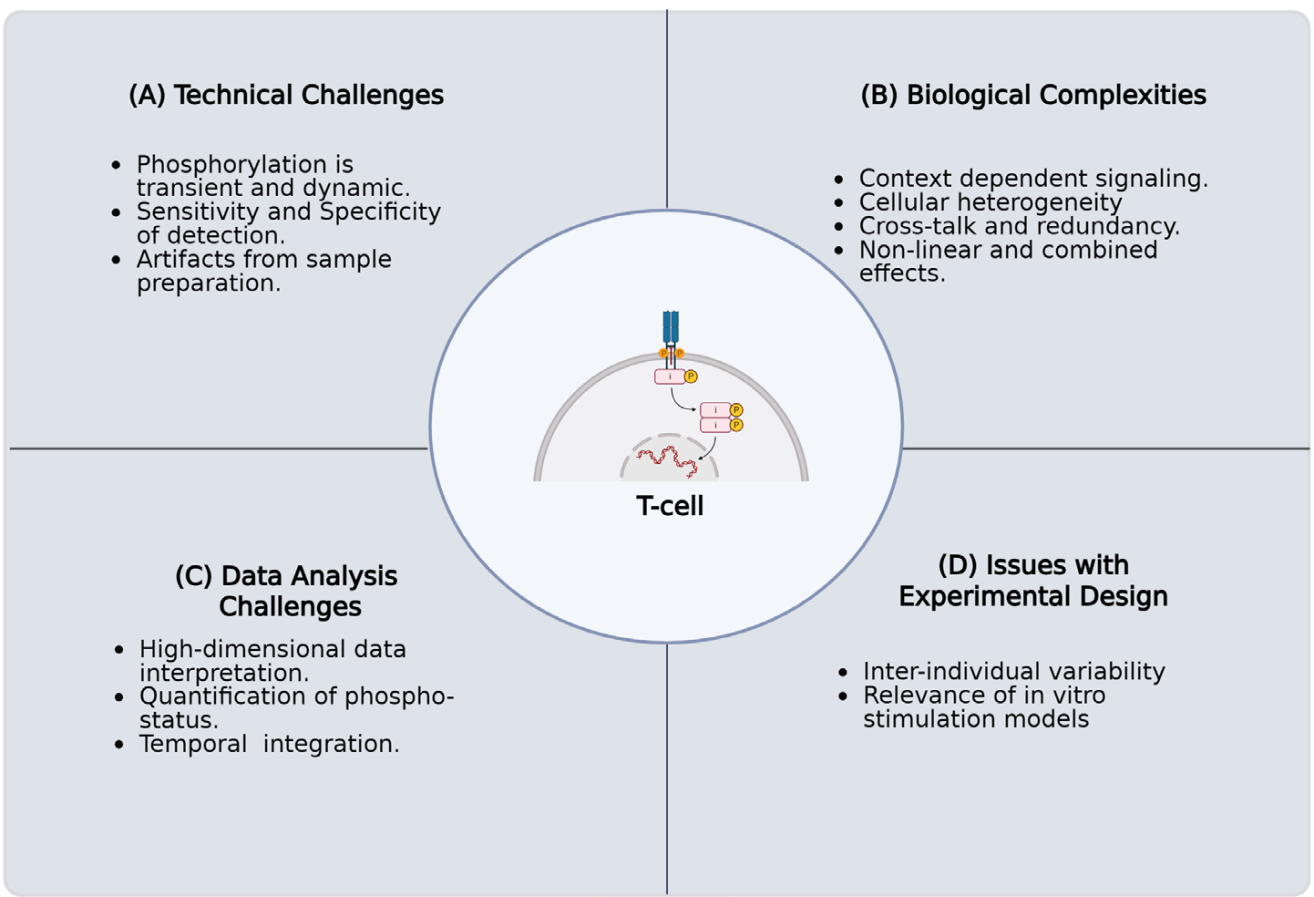
Challenges in characterizing protein phosphorylation and its impact on T-cell fate and function in primary human samples. The key challenges encountered when investigating the intricate role of protein phosphorylation in T-cell signaling pathways using primary human samples. These complications arise for several reasons. (**A**) Technical challenges involving the inherent biological complexity of T cells, including their diverse subsets, dynamic activation states, and rare antigen-specific populations. (**B**) Biological complexities, including context-dependent signaling, cellular heterogeneity, crosstalk, and nonlinear combined effects. (**C**) Challenges in high-dimensional data analysis, necessitated by single-cell resolution and multiomics approaches, present difficulties in data integration, normalization, noise reduction, and interpretation. (**D)** Issues with experimental design, such as execution, acquisition of sufficient numbers of quality primary human T cells, selecting appropriate controls, addressing donor variability, and confirming inter-experimental reproducibility. Overcoming these technical limitations is critical for a comprehensive understanding of T-cell biology and function.

T-cell signaling depends on protein phosphorylation to activate and regulate signaling cascades that influence T-cell activation, differentiation, and effector function. This process begins immediately when antigens presented by antigen-presenting cells (APCs) bind to the T-cell receptor (TCR). This binding results in the phosphorylation of the receptor's intracellular domain, triggering a series of phosphorylation events along multiple pathways, including the MAPK/ERK kinase (MEK), protein kinase C (PKC), nuclear factor-κB (NF-κB), and JAK/STAT pathways. Despite advances in understanding T-cell signaling, there has been limited progress in characterizing the role of protein phosphorylation in T-cell fate and function at the single-cell level. This limitation is primarily due to technical challenges, including but not limited to the biological complexity of T cells, the need for advanced analysis techniques to handle high-dimensional data, and complications in experimental design (see Figure 1). TCR activation is crucial for the effectiveness of T-cell-targeted immunotherapies; however, current assays are insufficient for fully elucidating the phosphorylation status of key targets downstream of the TCR in patient samples. Furthermore, leveraging technologies that enable high-dimensional analysis of TCR signaling at the single-cell level could yield important insights into disorders associated with impaired T-cell function.

TCR signaling is crucial for the activation, proliferation, and differentiation of naïve T cells into effector and memory subsets [3]. The activation of naïve T cells involves three key steps. First, an antigen is presented by the major histocompatibility complex (MHC) on antigen-presenting cells (APCs) and binds to the TCR, initiating a signaling cascade. This engagement also activates T-cell co-stimulatory receptors, which bind to their corresponding ligand receptors on APCs. For instance, the CD28 receptor on T cells interacts with the B7.1 or B7.2 ligands on APCs as a response to TCR activation. These interactions enhance T-cell activation and promote their proliferation. The third step is differentiation, which is driven by the secretion of autocrine and paracrine cytokines within the microenvironment. Together, these external signals influence intracellular processes that lead to changes in gene expression, ultimately shaping the T-cell response. Differential T-cell activation states can be investigated by analyzing intracellular protein phosphorylation following TCR stimulation. In patient samples with immunodeficiencies or those that have received T-cell therapies, understanding the depth of T-cell function can be achieved by examining phosphorylation patterns downstream of TCR signaling. However, little is known about how phosphorylation patterns vary in response to different T-cell activation states at the single-cell level. Therefore, developing a workflow to assess single-cell phosphorylation profiles of important signaling proteins after T-cell activation will be vital for defining the molecular mechanisms that drive T-cell function or dysfunction in patient samples.

Mass cytometry by time-of-flight (CyTOF) combines the principles of flow cytometry with mass spectrometry, making it a powerful, high-dimensional technique for single-cell protein analysis. This technology excels in assessing immunophenotypes and immune cell activation, making it integral to investigating immune status in complex human samples [4]. Unlike fluorescence-based flow cytometry, CyTOF employs heavy metal–tagged antibodies, typically from the lanthanide series, for protein detection. Upon acquisition, a sample is first nebulized and then introduced into an argon plasma, which vaporizes the cells and ionizes the metal tags into an ion cloud. Next, a time-of-flight mass spectrometer separates and measures the ions based on their mass-to-charge ratio. Each metal isotope is detected in a distinct channel, resulting in minimal or no overlap, remediating the issue of spectral overlap experienced with flow cytometric methods. This unique capability enables the simultaneous, high-resolution analysis of over 40 protein targets, including both intracellular and extracellular proteins, as well as those with post-translational modifications. CyTOF's increased capacity for target staining and minimal metal overlap enhances the likelihood of identifying rare or unexpected cell populations and capturing intracellular responses, even with limited sample material. Furthermore, this technology excels at characterizing immune cell activity within their native setting, revealing levels of heterogeneity, protein expression, and cellular complexity previously missed. Further, CyTOF enables the simultaneous investigation of differential signaling patterns across multiple pathways, such as those activated downstream of the TCR, which allows for the study of phosphorylation status. In comparison with alternative techniques, CyTOF excels in single-cell functional characterization of immune status through differential phosphorylation.

Fernandez and Maecker introduced the application of CyTOF to human blood samples in their *Bio-Protocol* publication, which describes a workflow for cytokine-stimulated phosphoflow of whole blood [1]. To achieve T-cell activation, the Stanford Human Immune Monitoring Core (HIMC) protocol, which utilizes an anti-CD3 antibody [5] and Protein A, was used as a benchmark for TCR activation. This method does not provide co-stimulation, which may lead to T-cell anergy and skewed results regarding T-cell activation. Currently, there is limited information in the literature regarding the in vitro stimulation of acute T-cell activation and the expected phosphorylation patterns in primary human T cells using endogenous stimuli that provide both required signals for activation. To enhance existing protocols, we utilized the framework established by Fernandez and Maecker [1] to develop a method for investigating T-cell activation and phosphorylation in response to more endogenous stimuli, specifically the combination of anti-CD3 antibody and anti-CD28 antibody, in whole blood. This approach may offer insights into the dynamics of T-cell function. Also, the use of "whole blood biopsies" as a less invasive method to study changes in the immune landscape longitudinally throughout disease progression and treatment can complement the spatial resolution provided by tissue analysis. In cases of immune dysregulation or cancer, high-dimensional techniques, such as a T-cell phospho-CyTOF pipeline, can assist in predicting viable treatment options and patient responses to therapeutic interventions. The ability to evaluate the functions of cytokine and antigen receptors, as well as alternative signaling pathways, in whole blood may yield valuable information about T-cell status with minimal invasiveness. Therefore, the innovation of technologies that enhance the throughput and resolution of these assays is essential.

## Materials and reagents

**Biological materials**

1. Human whole blood collected by venipuncture and preserved in sodium heparin (green top) tubes (NIH Clinical Center Blood Bank)

2. Jurkat, clone E6-1 (ATCC, catalog number: TIB-152)

**Reagents**

1. IFNα (PBL, catalog number: 11105-1)

2. Lipopolysaccharide (LPS) from *Salmonella enterica* serotype enteritidis, γ-irradiated, BioXtra, suitable for cell culture (Sigma-Aldrich, catalog number: L7770-1MG)

3. Anti-human CD3 (clone UCHT1) (BD Biosciences, catalog number: 555329)

4. Protein-A (Sigma-Aldrich, catalog number: P6031-1MG)

5. ImmunoCult Human CD3/CD28 T-cell activator (StemCell Technologies, catalog number: 10971)

6. RPMI 1640 + GlutaMax (Thermo Fisher Scientific, catalog number: 61870036)

7. Heat-inactivated fetal bovine serum (FBS) (Thermo Fisher Scientific, catalog number: A5670801)

8. Dulbecco’s modified PBS without Ca^2+^/Mg^2+^ (Thermo Fisher Scientific, catalog number: 14190094)

9. Penicillin-Streptomycin (Pen/Strep) (10,000 U/mL) (Thermo Fisher Scientific, catalog number: 15140122)

10. Proteomic Stabilizer 1 (Prot1) (SmartTube Inc., catalog number: 501351691)

11. Thaw/lyse concentrate (SmartTube Inc., catalog number: 501351696)

12. Methanol (MeOH) ACS reagent, ≥99.8%, 1 L (Sigma-Aldrich, catalog number: 179337-1L)

13. Water, molecular biology grade (Quality Biological, catalog number: 351-029-101)

14. Maxpar^®^ 10× Barcode perm concentrate (50 mL) (Standard BioTools, catalog number: 201057)

15. Maxpar^®^ PBS (500 mL) (Standard BioTools, catalog number: 201058)

16. Maxpar^®^ cell staining buffer (CSB) (500 mL) (Standard BioTools, catalog number: 201068)

17. Cell-ID^TM^ 20-Plex Pd Barcoding kit (Standard BioTools, catalog number: 201060)

18. Cell-ID^TM^ Intercalator-Ir, 12.5 μM (500 μL) (Standard BioTools, catalog number: 201192C)

19. EQ Four Element calibration beads (EQ4 beads) (100 mL) (Standard BioTools, catalog number: 201078)

20. Maxpar^®^ fix and perm buffer (100 mL) (Standard BioTools, catalog number: 201067)

21. Maxpar^®^ cell acquisition solution plus (CAS^+^) for CyTOF^®^ XT (1,000 mL) (Standard BioTools, catalog number: 201244)

22. Maxpar^®^ water (500 mL) (Standard BioTools, catalog number: 201069)

23. CyTOF tuning solution (250 mL) (Standard BioTools, catalog number: 201072)

**Solutions**

1. Stimulation media (cRPMI) (see Recipes)

2. 1× Thaw/lyse buffer (see Recipes)

3. 1× Barcoding (BC) buffer (see Recipes)

4. Iridium intercalation solution (see Recipes)

5. 1:10 EQ4 beads (see Recipes)

**Recipes**

**1. Stimulation media (cRPMI)**

**Table:** 

Reagent	Final concentration	Volume
RPMI 1640 + GlutaMax	89%	445 mL
Heat-inactivated FBS	10%	50 mL
Pen/strep	1%	5 mL
Total		500 mL

Filter-sterilize the stimulation media through a 500 mL 0.22 μm filtration unit. Stimulation media can be prepared up to one month before use and stored at 4 °C.

**2. 1× Thaw/lyse buffer**

**Table:** 

Reagent	Final concentration	Volume
Molecular biology–grade water		49.95 mL
Thaw/lyse concentrate (1,000×)	1×	0.05 mL
Total		50.00 mL

Filter-sterilize the 1× thaw/lyse buffer through a 500 mL 0.22 μm filtration unit. 1× thaw/lyse buffer can be prepared and stored at room temperature (RT) for up to one month from the preparation date.

**3. 1× Barcoding (BC) buffer**

**Table:** 

Reagent	Final concentration	Volume
MaxPar PBS		45mL
Maxpar^®^ 10× barcode perm concentrate	1×	5 mL
Total		50 mL

1× barcoding buffer can be stored at 4 °C and used for up to one month from the preparation date.

**4. Iridium intercalation solution**

**Table:** 

Reagent	Final concentration	Volume
Maxpar^®^ fix and perm buffer		3.998 mL
Cell-ID^TM^ Intercalator-Ir, 12.5 μM	2,000×	0.002 mL
Total		4.0 mL

Safe handling of iridium requires strict adherence to lab safety protocols for heavy metals. Specific iridium compounds are toxic, corrosive, or irritating. Wear appropriate personal protective equipment and work in a fume hood when applicable.

Cell-ID^TM^ intercalator-Ir is light-sensitive. Ensure that any aliquots or solutions are protected from light.

Cell-ID^TM^ intercalator-Ir should be aliquoted and frozen at -20 °C immediately after thawing. After a second thaw, aliquots of Cell-ID^TM^ intercalator-Ir should be discarded.

The iridium intercalation solution should be prepared immediately before use and stored on wet ice.

**5. 1:10 EQ4 beads**

**Table:** 

Reagent	Final concentration	Volume
Maxpar^® ^Cell acquisition solution plus (CAS^+^) for CyTOF XT		9 mL
EQ Four Element calibration beads	10×	1 mL
Total		10 mL

The EQ Four Element calibration beads require vigorous shaking by hand for at least 30 s prior to use. Do not vortex the EQ4 beads, as vortexing fails to resuspend the beads into solution. The 1:10 EQ4 beads in CAS^+^ should be prepared immediately before use and stored on ice.

**Laboratory supplies**

1. 25 mL sterile reservoirs (Thermo Fisher Scientific, catalog number: 95128095)

2. DNA LoBind tube 2.0 mL (Eppendorf, catalog number: 022431048)

3. Safe-Lock tubes 1.5 mL, natural (Eppendorf, catalog number: 022363212)

4. Matrix 0.5 mL 2D screw tubes PP, V-bottom, w/ clear caps (Thermo Fisher Scientific, catalog number: 3744)

5. Matrix 0.5 mL 2D screw tubes PP, V-bottom, w/ yellow caps (Thermo Fisher Scientific, catalog number: 3744YEL)

6. Matrix 0.5 mL 2D screw tubes PP, V-bottom, w/ blue caps (Thermo Fisher Scientific, catalog number: 3744BLU)

7. Matrix 0.5 mL 2D screw tubes PP, V-bottom, w/ red caps (Thermo Fisher Scientific, catalog number: 3744RED)

8. Matrix 0.5 mL 2D screw tubes PP, V-bottom, w/ pink caps (Thermo Fisher Scientific, catalog number: 3744PIN)

9. 14 mL polystyrene round-bottom tube (Corning, catalog number: 352051)

10. 5 mL polystyrene round-bottom tube 12 × 75 mm style (Falcon, catalog number: 352058)

11. 5 mL polystyrene round-bottom tube with cell strainer cap (Falcon, catalog number: 352235)

12. 50 mL polypropylene conical tube 30 × 115 mm style (Corning, catalog number: 352070)

13. LTS 1,200 μL filter 768/4 tips (Rainin, catalog number: 17007084)

14. TR LTS 1,000 μL filter 768/4 tips (Rainin, catalog number: 17014967)

15. Pipette tips RT LTS 300 μL 768A/8 (Rainin, catalog number: 30389253)

16. RTS LTS 200 μL F 960A/10 (Rainin, catalog number: 30389239)

17. Dualfilter 200 μL, PCR clean/sterile (Eppendorf, catalog number: 022491296)

18. P20 LTS pipette tips (Rainin, catalog number: 17014392)

19. Sterile 5 mL serological pipettes (VWR Scientific, catalog number: 170355N)

20. Sterile 10 mL serological pipettes (VWR Scientific, catalog number: 89130-898)

21. Nunc plate seals (ThermoFisher Scientific, catalog number: AB0718)

22. 96-well deep well 2 mL plate (Fisher Brand, catalog number: 12-566-121)

23. 96-well V-bottom plate with lid polystyrene TC-treated (Corning, catalog number: 3894)

24. 96-well U-bottom plate with lid polystyrene TC-treated (Corning, catalog number: 3799)

25. C-Chip (inCyto, catalog number: DHC-N01-5)

## Equipment

1. Helios CyTOF (Standard BioTools, catalog number: PN 400250 A7)

2. Thermo Sorvall Legend XTR Refrigerated Centrifuge (Marshall Scientific, catalog number: TSO-LEGXTR)

3. Corning^TM^ Mini Microcentrifuge, 100–240 V (Fisher Scientific, catalog number: 07-203-954)

4. -86 °C upright freezer (Phcbi, catalog number: MDFDU702VHPA)

5. -20 °C K2 Scientific Laboratory Undercounter Freezer (Fisher Scientific, catalog number: K204SDF)

6. 4 °C TSX Series High-Performance Lab Refrigerator (ThermoFisher Scientific, catalog number: TSX3005SD)

7. Eppendorf^TM^ Centrifuge 5430 R, Refrigerated Microcentrifuge (Fisher Scientific, catalog number: 13-690-005)

8. Water bath (Corning, catalog number: 6783)

9. Pipet-Lite Pipette, Universal SL-1000XLS+ (Mettler Toledo, catalog number: 17014407)

10. Pipet-Lite Pipette, Universal SL-200XLS+ (Mettler Toledo, catalog number: 17014391)

11. Pipet-Lite LTS Pipette L-20XLS+ (Mettler Toledo, catalog number: 17014392)

12. Pipet-Lite LTS Pipette L-10XLS+ (Mettler Toledo, catalog number: 17014388)

13. Pipet-Lite LTS Pipette L-2XLS+ (Mettler Toledo, catalog number: 17014393)

14. Pipet-Lite Pipette Multi L12-1200XLS+ (Mettler Toledo, catalog number: 17014497)

15. Pipet-Lite Multi Pipette L12-300XLS+ (Mettler Toledo, catalog number: 17013811)

16. Pipet-Lite Multi Pipette L12-200XLS+ (Mettler Toledo, catalog number: 17013810)

17. Corning^TM^ 8-Channel Adapter for Vacuum Aspirator (Corning, catalog number: 4931)

18. Pipet-Aid^®^ XP (Drummond Scientific Company, catalog number: 4-000-101)

19. Eppendorf^®^ Thermomixer^®^ FP (Millipore Sigma, catalog number: EP5385000024)

20. Vortex Genie-2 (Millipore Sigma, catalog number: Z258423)

21. Forma^TM^ Series II Water-Jacketed CO2 Incubator (Thermo Fisher, catalog number: 3131)

22. 1300 Series Class II, Type A2 Biological Safety Cabinet Packages (Thermo Fisher, catalog number: 1323TS)

23. Microscope (Olympus, catalog number: CKX53)

## Software and datasets

1. CyTOF Software (Standard BioTools, version 7.1), https://www.fluidigm.com/software

2. Excel (Microsoft, version 2408), license required, https://www.microsoft.com/en-us/microsoft-365/excel

3. FlowJo (TreeStar, version 10.10), license required, https://www.flowjo.com/solutions/flowjo/downloads/

4. GraphPad Prism (GraphPad Software LLC., version 9.5.0), license required, https://www.graphpad.com/scientific-software/prism/

5. RStudio (Posit Software, PBC, version 2024.09.1+394), no license required, https://posit.co/download/rstudio-desktop/

6. HDStIM (GitHub) [6], no license required, https://doi.org/10.1101/2022.11.14.516371

## Procedure

**A. Barcode preparation and pooling (optional) (30 min)**

*Notes:*

*1. Barcodes for whole blood samples can be prepared the day before processing.*

*2. A single barcode ampule is sufficient for two wells of whole blood. For example, two ampules of each barcode BC1-15 were thawed to barcode four whole blood samples.*

*3. Refer to Table 3.*

1. Remove the appropriate barcode ampules from -20 °C and thaw on wet ice.

2. Following the plate layout (see Table 3), remove enough ampules to provide 50 μL of barcodes per well.

3. Resuspend each ampule of barcodes (BC 1-15) with 100 μL of 1× BC buffer.

4. Transfer the contents of each barcode ampule into a single well of a 96-well U-bottom plate.

5. Do not exceed two ampules of the same barcode in any one well.

6. Store the 96-well U-bottom plate with the resuspended barcodes at 4 °C until ready for use.

**B. Preparation of stimulation stocks and matrix tubes (1–2 h)**

*Notes:*

*1. Each matrix tube cap color is assigned to a particular stimulation (stimuli).*

*2. Refer to Table 2 for an example of how different stimuli are color-coded for banking and storage at -80 °C.*

1. Label 0.5 mL color-coded matrix tubes in accordance with the number of samples and replicates.

2. Prepare all stimulation conditions at 5× concentrations in stimulation media using sterile 1.5 mL Eppendorf tubes. Ensure there is enough volume to aliquot 50 μL into each matrix tube. Refer to the example in Tables 1 and 3 for four whole blood donors, with six replicates of each stimulation condition.

3. For the HIMC stimulation, combine 5× anti-human CD3 with 5× Protein-A.

4. Aliquot 50 μL of each 5× stimulation stock to the colored matrix tube representative of the stimulation condition banked for that colored tube. For example, 50 μL of 5× IFNα stock is aliquoted into blue-capped matrix tubes.

5. The matrix tubes containing stimulation reagents can be stored frozen at -80 °C until needed.

*Note: Scale up the volume of stimulation solutions (stimuli) prepared based on the number of donors and replicates that will be banked.*

**Table 1.** Example stimulation preparation

Stimulation	[Stock]	Stimulant [1×]	Stimulant [5×]	5× stimulant (μL)	cRPMI (μL)	Total volume (μL)
UNS	Stimulation media	NA	NA	NA	NA	1,500.000
IFNα	2.09E+06 IU/mL	10,000 IU/mL	50,000 IU/mL	34.988	1,427.512	1,462.500
LPS	500 μg/mL	1 μg/mL	5 μg/mL	15.000	1,485.000	1,500.000
Anti-human CD3	1,000 μg/mL	10 μg/mL	50 μg/mL	75.000	1,350.000	1,425.000
Protein-A	1,000 μg/mL	10 μg/mL	50 μg/mL	75.000	1,425.000	1,500.000
StemCell activator	50 μL/test			50.000	NEAT	NEAT

The HIMC stock solution is the combination of anti-human CD3 (cl. UCHT1) and Protein-A, which is prepared in a volume equating to 1,500 μL.

To prepare the HIMC stock solution, aliquot 75 μL of anti-human CD3 (cl. UCHT1) and 75 μL of Protein-A into 1,350 μL of cRPMI.

**Table 2.** Example matrix tube color coding by stimulation

Matrix tube color	Associated (5×) stimulation stock solution
Clear	Unstimulated (UNS)
Yellow	Anti-human CD3 (cl. UCHT1) + Protein-A (HIMC)
Blue	IFNα (IFA)
Red	StemCell activator (SC)
Pink	Lipopolysaccharide (LPS)

**C. Whole blood stimulation and cryopreservation (~1.5 h)**

**Table 3.** Example plate layout for whole blood lysis and barcoding

UNS WB1 Rep1 (BC1)	HIMC WB1 Rep1 (BC2)	IFA WB1 Rep1 (BC3)	SC WB1 Rep1 (BC4)	LPS WB1 Rep1 (BC5)	**BC Set 1**	UNS WB2 Rep1 (BC1)	HIMC WB2 Rep1 (BC2)	IFA WB2 Rep1 (BC3)	SC WB2 Rep1 (BC4)	LPS WB2 Rep1 (BC5)	**BC Set 2**
UNS WB1 Rep2 (BC6)	HIMC WB1 Rep2 (BC7)	IFA WB1 Rep2 (BC8)	SC WB1 Rep2 (BC9)	LPS WB1 Rep2 (BC10)		UNS WB2 Rep2 (BC6)	HIMC WB2 Rep2 (BC7)	IFA WB2 Rep2 (BC8)	SC WB2 Rep2 (BC9)	LPS WB2 Rep2 (BC10)	
UNS WB1 Rep3 (BC11)	HIMC WB1 Rep3 (BC12)	IFA WB1 Rep3 (BC13)	SC WB1 Rep3 (BC14)	LPS WB1 Rep3 (BC15)		UNS WB2 Rep3 (BC11)	HIMC WB2 Rep3 (BC12)	IFA WB2 Rep3 (BC13)	SC WB2 Rep3 (BC14)	LPS WB2 Rep3 (BC15)	
											
UNS WB3 Rep1 (BC1)	HIMC WB3 Rep1 (BC2)	IFA WB3 Rep1 (BC3)	SC WB3 Rep1 (BC4)	LPS WB3 Rep1 (BC5)	**BC Set 3**	UNS WB4 Rep1 (BC1)	HIMC WB4 Rep1 (BC2)	IFA WB4 Rep1 (BC3)	SC WB4 Rep1 (BC4)	LPS WB4 Rep1 (BC5)	**BC Set 4**
UNS WB3 Rep2 (BC6)	HIMC WB3 Rep2 (BC7)	IFA WB3 Rep22 (BC8)	SC WB3 Rep2 (BC9)	LPS WB3 Rep2 (BC10)		UNS WB4 Rep2 (BC6)	HIMC WB4 Rep2 (BC7)	IFA WB4 Rep2 (BC8)	SC WB4 Rep2 (BC9)	LPS WB4 Rep2 (BC10)	
UNS WB3 Rep3 (BC11)	HIMC WB3 Rep3 (BC12)	IFA WB3 Rep3 (BC13)	SC WB3 Rep3 (BC14)	LPS WB3 Rep3 (BC15)		UNS WB4 Rep3 (BC11)	HIMC WB4 Rep3 (BC12)	IFA WB4 Rep3 (BC13)	SC WB4 Rep3 (BC14)	LPS WB4 Rep3 (BC15)	

Whole blood is transferred and lysed in a 2 mL deep-well plate. The lysed blood samples are transferred from the 2 mL deep-well plate to a sterile 96-well V-bottom plate in the same order as above for barcoding (**Section E**).

1. Prepare a detailed plate layout for whole blood lysis and barcoding (refer to Table 3).

2. Carefully collect whole blood from the designated donor(s) using venipuncture.

3. Immediately transfer the collected blood into sodium heparin (green top) tubes to prevent clotting.

4. Ensure that blood collection is performed under aseptic conditions to maintain sample integrity.

5. Place the sodium heparin tubes containing the whole blood into a 37 °C incubator with 5% CO_2_.

6. Allow the tubes to equilibrate in the 37 °C incubator with 5% CO_2_ for 30 min to 1 h.

7. Remove the matrix tubes containing the stock stimulation solutions from -80 °C 20 min before the stimulation cocktail is intended for use.

8. Thaw the matrix tubes in a 37 °C 5% CO_2_ incubator or in a water bath at 37 °C.

9. For each donor sample, ensure that one matrix tube of each stimulation stock solution is thawed for every replicate that will be processed.

10. Working in a laminar flow hood, aliquot 200 μL of the whole blood from each tube into the corresponding matrix tubes, adhering to the pre-designed plate layout.

11. Mix the matrix tube contents thoroughly by pipetting up and down 10×.

12. Work quickly and change pipette tips between each transfer to maintain sample purity and prevent cross-contamination.

13. Tap matrix tubes gently to bring the contents of each tube to the bottom.

14. Incubate the matrix tubes in their sealed holder for 15 min at 37 °C in the 5% CO_2_ incubator.

15. Add 250 μL of Prot1 to each matrix tube in the same order as the whole blood was added.

16. Using a P300 multichannel pipette, mix each tube 10×, changing the tips between each tube to prevent contamination.

17. Incubate the matrix tubes at RT for 10 min.

**Pause point:** Stimulated and fixed whole blood can be frozen at -80 °C until it is ready for thawing, processing, and CyTOF analysis.

**D. Whole blood lysis (2 h)**

*Notes:*

*1. Immediately proceed to step D2 if samples were not frozen after stimulation.*

*2. The matrix tubes containing stimulated and fixed whole blood should only be thawed for lysis if all samples can be run on the CyTOF within 1 day (maximum of 60 matrix tubes per run).*

1. Thaw the matrix tubes to a 25 °C water bath for 15 min.

2. Transfer 500 μL of the stimulated and fixed whole blood from each matrix tube into a 2 mL deep-well plate containing 1.2 mL of 1× thaw/lyse buffer.

3. Mix thoroughly using a P1200 multichannel pipette.

4. Seal the deep-well plate using a plate sealer.

5. Allow the whole blood samples to lyse for 10 min at RT.

6. Centrifuge the deep well containing the lysed whole blood at 548× *g* for 10 min at RT.

7. Carefully remove the supernatant with a 12-well multichannel, changing tips between each well.

8. Seal the top of the deep-well plate using a plate sealer.

9. Gently vortex the sealed plate for 30 s or until the cell pellets are dispersed.

10. Add 1.6 mL of 1× thaw/lyse buffer and mix thoroughly using a P1200 multichannel pipette.

11. Allow the whole blood sample to lyse for 10 min at RT.

12. Centrifuge the deep well containing the lysed whole blood at 548× *g* for 10 min at RT.

13. Carefully remove the supernatant with a 12-well multichannel, changing tips between each well.

14. Seal the top of the deep-well plate using a plate sealer and gently vortex the pellet.

15. If substantial RBCs remain, repeat steps D10–13. If not, wash the pellet with 1.6 mL of cell staining buffer (CSB).

*Note: Do not exceed three lysis steps.*

16. Centrifuge cells at 974× *g* for 10 min at 4 °C.

17. Carefully remove the supernatant with a 12-well multichannel, changing tips between each well.

18. Leave 50 μL at the bottom of each well.

19. Resuspend each well with 100 μL of CSB.

20. Transfer 150 μL of cell suspension into a 96-well V-bottom plate and proceed to the barcoding or staining step.

**E. Barcoding (optional) (1.5 h)**

1. Centrifuge the 96-well V-bottom plate containing the cell suspensions at 974× *g* for 5 min at 4 °C.

2. Flick the supernatant and blot the plate on a clean paper towel.

3. If any liquid remains in the deep-well plate from step D20, transfer the remaining volume into the 96-well V-bottom plate in the same order as initially added.

4. Centrifuge the 96-well V-bottom plate at 974× *g* for 5 min at 4 °C.

5. Flick the supernatant from the plate and blot on a clean paper towel to remove residual liquid.

6. Resuspend cells in 200 μL of ice-cold 1× BC buffer and centrifuge at 974× *g* for 5 min at 4 °C.

7. Flick the supernatant from the plate and blot the plate on a clean paper towel to remove residual liquid.

8. Resuspend each well in 50 μL of the appropriate barcode (refer to Table 3).

9. Incubate the 96-well V-bottom plate on the thermomixer at 25 °C with 350 rpm shaking for 30 min.

10. Add 140 μL of ice-cold 1× BC buffer to all wells.

11. Centrifuge the 96-well V-bottom at 974× *g* for 5 min at 4°C.

12. Flick the supernatant from the plate and blot on a clean paper towel to remove residual liquid.

13. Resuspend the cells in 100 μL of CSB and combine the appropriate wells (up to 15 barcoded samples should be combined into one tube) into an appropriately labeled 2 mL Eppendorf tube (BC Set 1–4, refer to Table 3 for labeling of 2 mL Eppendorf tubes for barcoded sets).

14. Rinse each well with 100 μL of CSB and collect the contents into a second set of 2 mL Eppendorf tubes labeled as wash tubes (BC 1–4 Wash, refer to Table 3 for labeling of wash tubes for each barcoded set).

15. Centrifuge all tubes at 1,000× *g* in the microfuge for 5 min at 4 °C.

16. Carefully aspirate the supernatant without dislodging the pellets.

17. Pool the primary and wash tubes by resuspending each tube in 500 μL of CSB and transferring the contents of the wash tube to the primary tube.

18. Add 800 μL of CSB to the primary tube.

19. Centrifuge the tubes at 1,000× *g* for 5 min at 4 °C.

20. Carefully aspirate the supernatant without dislodging the pellet.

21. Measure the residual volume in each 2 mL Eppendorf.

22. If the volume is less than 50 μL per barcode sample, add enough CSB to ensure the final volume is equivalent to (# barcodes × 50 – residual volume) (i.e., 750 μL: residual volume for 15 barcodes).

**F. Surface staining and methanol permeabilization (2–3 h)**

1. Prepare the 2× surface master mix (refer to Table 4).

**Table 4.** Surface master mix for immunophenotyping of whole blood (example for 60 samples)

Metal	Target	Vendor	Catalog number	Clone	Ab (μL)
^141^Pr	CD7	BioLegend	343111	CD7-6B7	9
^151^Eu	CD123 (IL-3R)	Standard BioTools	3151001B	6H6	9
^155^Gd	CD27	Standard BioTools	3155001B	L128	9
^159^Tb	CD11c	Standard BioTools	3159001B	Bu15	9
^168^Er	CD8a	Standard BioTools	3168002B	SK1	9
^176^Yb	CD127 (IL-7Ra)	Standard BioTools	3176004B	A019D5	18
^142^Nd	CD19	Standard BioTools	3142001B	HIB19	18
^157^Gd	CD24	BioLegend	311127	ML-5	18
^167^Er	CD38	Standard BioTools	3167001B	HIT2	18
^170^Er	CD3	Standard BioTools	3170001B	UCHT1	18
^174^Yb	HLA-DR	Standard BioTools	3174001B	L243	18
^209^Bi	CD16	Standard BioTools	3209002B	3G8	36
^145^Nd	CD4	Standard BioTools	3145001B	RPA-T4	36
^160^Gd	CD14	Standard BioTools	3160006B	RMO52	36
^147^Sm	CD20	Standard BioTools	3147001B	2H7	36
^162^Dy	CD66b	Standard BioTools	3162023B	80H3	72
^89^Y	CD45	Standard BioTools	3089003B	HI30	45
^146^Nd	IgD	Standard BioTools	3146005B	IA6-2	45
^149^Sm	CD25 (IL-2R)	Standard BioTools	3149010B	2A3	45
^163^Dy	CD56	Standard BioTools	3163007B	NCAM16.2	22.5
^169^Tm	CD45RA	Standard BioTools	3169008B	HI100	11.25
Volume of cell staining buffer (CSB)					3,062.25

The surface master mix is made in CSB to account for a 20% excess volume. For example, 60 samples require 3,600 μL of surface master mix.

Prepare 1:10 dilutions of anti-CD45, anti-IgD, anti-CD25 (IL-2R), anti-CD56, and anti-CD45RA in CSB and use the 10× antibody stocks to make the surface master mix. These antibody volumes have been used to stain up to 4 million cells.

2. Add 50 μL of 2× surface master mix per barcoded sample to each tube (15 pooled barcoded samples would use 750 μL of the surface master mix).

3. Incubate the Eppendorf tubes on the thermomixer at 25 °C with 350 rpm shaking for 30 min.

4. Centrifuge at 1,000× *g* for 5 min at 4 °C and aspirate the supernatant.

5. Resuspend in 1.8 mL of CSB.

6. Centrifuge at 1,000× *g* for 5 min at 4 °C.

7. Carefully aspirate the supernatant without dislodging the pellet.

8. Repeat steps F5–7 once.

9. Add 800 μL of ice-cold 100% MeOH dropwise, followed by an additional 1 mL of 100% MeOH.

**Pause point:** Samples can be stored at -80 °C overnight in 100% methanol to facilitate the workflow. If required, samples can be stored for 4–6 weeks in methanol.

*Note: If samples require intracellular staining, proceed to Section G.*

**G. Intracellular staining and iridium intercalation (2–3 h)**

1. Remove the Eppendorf tubes from the -80 °C freezer and transfer them to wet ice for 30 min.

2. Centrifuge at 1,000× *g* for 10 min at 4 °C.

3. Carefully aspirate the supernatant without dislodging the pellet.

4. Resuspend in 1.8 mL of CSB and centrifuge at 1,000× *g* for 5 min at 4 °C.

5. Carefully aspirate the supernatant without dislodging the pellet.

6. Repeat steps G4–5 once.

7. If the volume is less than 50 μL per barcode sample, add enough CSB to ensure the final volume is equivalent to (# barcodes × 50 – residual volume) (i.e., 750 μL: residual volume for 15 barcodes).

8. Prepare the 2× intracellular master mix (refer to Table 5).

**Table 5.** Intracellular master mix for phospho-analysis of stimulated whole blood (example for 60 samples)

Metal	Target	Vendor	Catalog number	Clone	Ab (μL)
^144^Nd	pPLCg2 (pY759)	Standard BioTools	3144015A	K86-689.37	9
^165^Ho	pCREB (S133)	Standard BioTools	3165034D	87G3	9
^175^Lu	pS6 (S235/S236)	Standard BioTools	3175009A	N7-548	9
^156^Gd	pP38 [T180/Y182]	Standard BioTools	3156002A	D3F9	18
^171^Yb	pERK 1/2 [T202/Y204]	Standard BioTools	3171010A	D13.14.4E	18
^152^Sm	pAkt (S473)	Standard BioTools	3152005A	D9E	36
^153^Eu	pSTAT1 [Y701]	Standard BioTools	3153003A	58D6	36
^158^Gd	pSTAT3 [Y705]	Standard BioTools	3158005A	4/P-STAT3	36
^150^Nd	pSTAT5 [Y694]	Standard BioTools	3150005A	47	36
^164^Dy	IKβα	Standard BioTools	3164004A	L35A5	36
Volume of cell staining buffer (CSB)					3,357.00

The intracellular master mix is made in CSB to account for a 20% excess volume. For example, 60 samples require 3,600 μL of intracellular master mix. These antibody volumes have been used to stain up to 4 million cells.

9. Add a volume of 2× intracellular master mix to each Eppendorf tube, equating to 50 μL per barcoded sample (15 pooled barcoded samples would require 750 μL of 2× intracellular mix).

10. Incubate the Eppendorf tube in the thermomixer at 25 °C with 350 rpm shaking for 30 min.

11. Centrifuge at 1,000× *g* for 5 min at 4 °C and aspirate the supernatant.

12. Resuspend in 1.8 mL of CSB, centrifuge at 1,000× *g* for 5 min at 4 °C, and aspirate supernatant.

13. Repeat step G12 once.

14. Prepare enough volume of 1:2,000 iridium intercalation solution to account for 100 μL per barcoded sample.

15. Resuspend cell pellets in freshly prepared iridium intercalation solution.

16. Store at 4 °C for a minimum of 30 min.

**Pause point:** Samples can be stored at 4 °C in iridium intercalation solution overnight. Do not exceed this duration.

**H. Sample preparation for Helios acquisition (6–10 h)**

1. Start up the Helios ~1 h:

a. Start-up plasma (during this time, process samples as indicated below) ~20 min.

b. Install nebulizer and check nebulizer spray ~2–5 min.

c. Check background ~1–2 min.

d. Tune ~16 min.

e. Run EQ4 beads ~2 min.

f. Run CAS^+^ buffer for a minimum of 15 min to precondition the wide-bore injector.

2. Retrieve samples from the 4 °C and mix by vortexing.

3. Add enough CSB to each 2 mL Eppendorf tube to bring the total volume to 1.8 mL.

4. Centrifuge at 1,000× *g* for 5 min at 4 °C.

5. Carefully aspirate the supernatant without dislodging the pellet.

6. Resuspend cells in 1.8 mL of CSB.

7. Centrifuge at 1,000× *g* for 5 min at 4 °C.

8. Carefully aspirate the supernatant without dislodging the pellet.

9. Repeat steps H6–8 once.

10. **Pause point:** Aspirate all but 50 μL of CSB from each tube.

*Notes:*

*1. Process each Eppendorf Tube one at a time.*

*2. When you cannot run all samples on the same day, centrifuge and remove all but 50 μL of CSB from the 2 mL Eppendorf tube. Store samples at 4 °C, sealed with parafilm to prevent evaporation.*

*3. These samples should be run as soon as possible, preferably the next day.*

**Caution:** Only samples that can be run in one day should be processed from CSB into CAS^+^ to avoid cell loss.

11. Resuspend cells in 1.8 mL of CAS^+^.

12. Centrifuge at 1,000× *g* for 10 min at 4 °C.

13. Carefully aspirate the supernatant without dislodging the pellet.

14. Resuspend cells in 1.8 mL of CAS^+^.

15. Repeat steps H11–13 once more.

16. Resuspend cells in 1 mL of freshly prepared 1:10 EQ4 beads diluted in CAS^+^.

17. Place the samples on ice.

18. Remove 10 μL for counting using a C-chip hemocytometer on the microscope.

19. Adjust the concentration of cells to 1.3 × 10^6^ cells/mL in 1:10 EQ4 beads diluted in CAS^+^. Aim to achieve an event rate of ~350–400 events/s (± 50 events/s).

*Note: The final volume for resuspension is dependent on the cell count from step H18.*

20. Proceed to run cells on the Helios mass cytometer.

**Figure 2.**
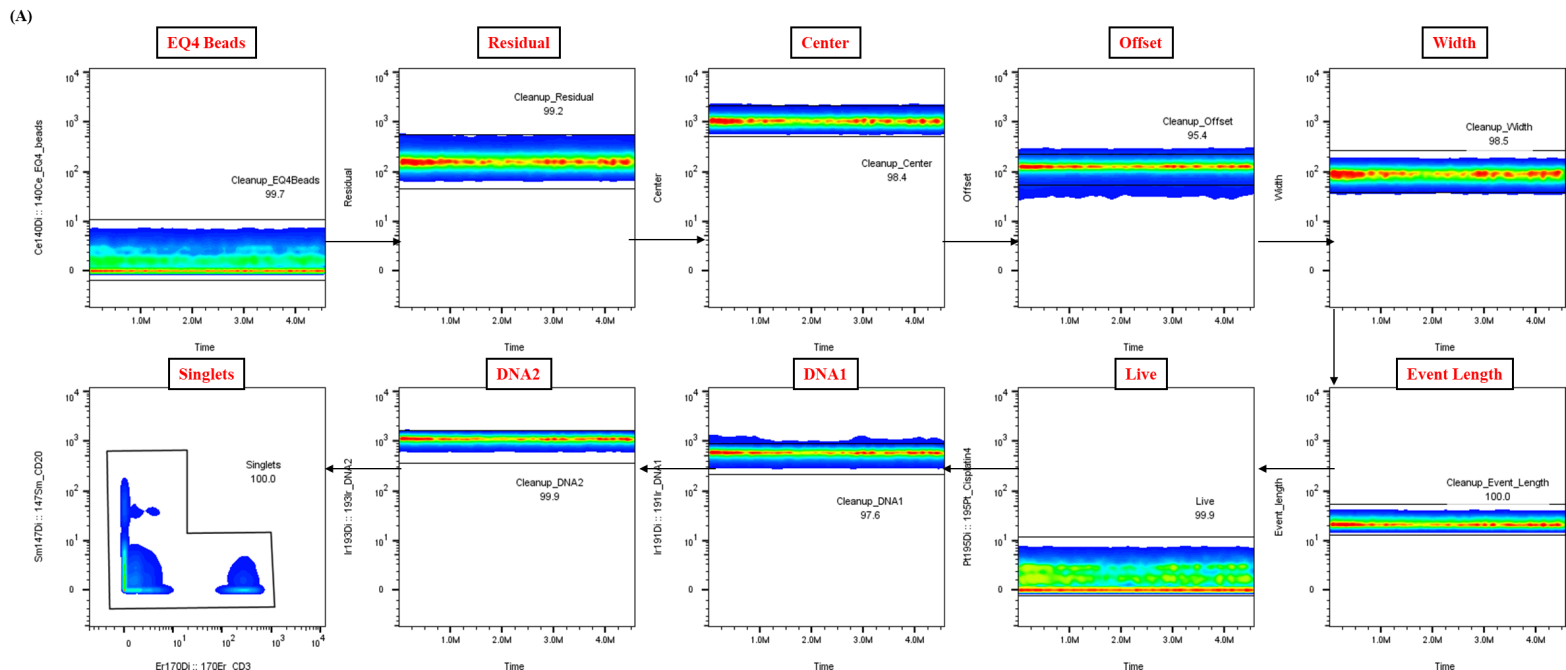
Representative data cleanup using Gaussian parameters for mass cytometry analysis. Representative example of the data cleanup strategy utilizing Gaussian parameters, a critical step in the analysis pipeline for healthy whole blood samples. The presented data were derived from a single control donor and illustrate the expected outcome of this cleanup process, essential for ensuring data quality and reproducibility.

**Figure 3.**
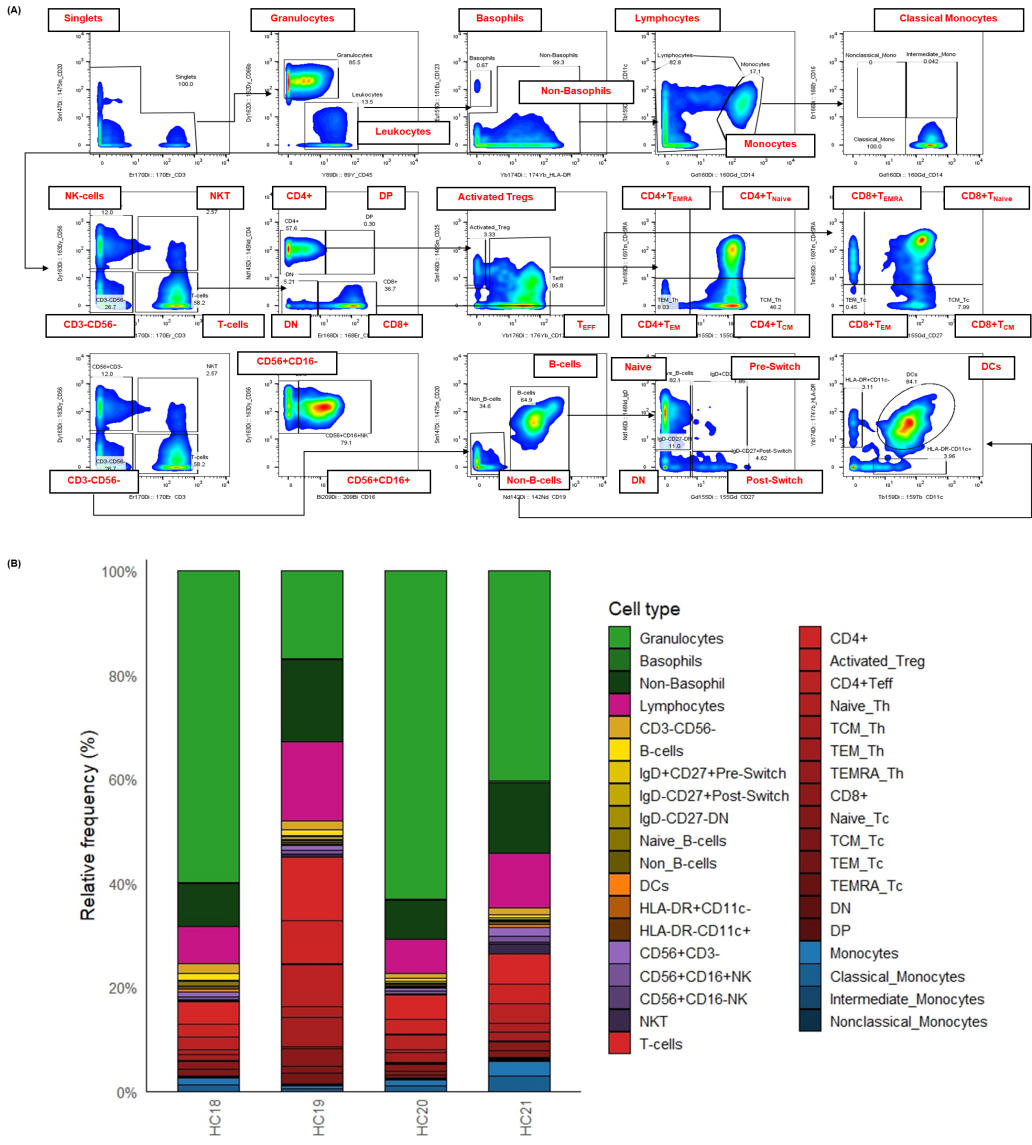
Surface immunophenotyping and relative frequencies of immune cell subsets in healthy human whole blood. This figure details the strategy for identifying major immune cell subsets in healthy human whole blood using a 21-marker surface panel with CyTOF. To facilitate the analysis of cellular composition, whole blood samples were fixed, barcoded, and stained according to the established protocol. Single cells were defined after several cleanup steps (as detailed in Figure 2) and the exclusion of doublets based on CD3 and CD20 expression. Subsequent gating was performed as follows: non-basophil leukocytes were identified as CD66b-CD45^+^. T cells (CD3^+^CD56-), NKT cells (CD3^+^CD56^+^), and total NK cells (CD3-CD56^+^) were distinguished using CD3 and CD56. B cells were defined as CD123-CD3-CD56-CD11c-CD19^+^CD20^+^. Further subsetting of T and B cells was achieved using additional markers, including CD4, CD8, CD45RA, CD27, CD25, CD127, and IgD. Non-T and non-B cells (CD45^+^CD3-CD19-CD56-CD20-) were gated as monocytes (CD14^+/-^, CD11c^+^, CD16^+/-^), dendritic cells (HLA-DR^+^CD11c^+^), and basophils (CD123^+^). (A) Bivariate plots illustrate the gating strategy used to identify major immune cell subsets in a representative healthy donor by CyTOF. (B) The stacked bar chart visualizes the relative frequency (% of total) of over 30 distinct cell populations defined by the expression of 21 surface markers from four (n = 4) healthy donors. Each stacked bar represents the average relative frequency of each immune subset of three biological replicates. This detailed immunophenotyping reveals the inherent variability in immune cell composition among healthy individuals.

**Figure 4.**
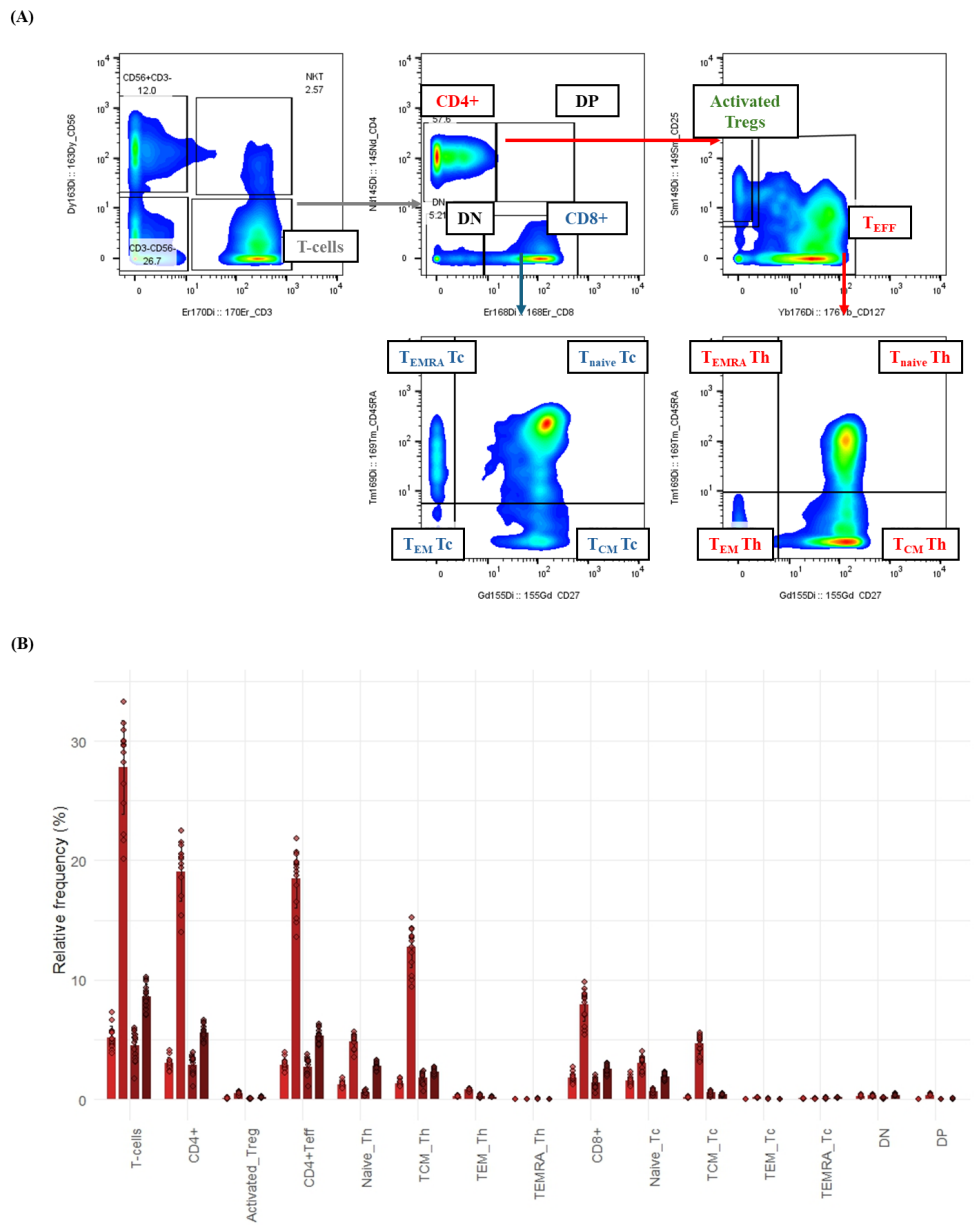
T-cell subsetting and composition in healthy whole blood by mass cytometry. This figure illustrates the detailed gating strategy for identifying T-cell subsets in healthy human whole blood using a 21-surface marker immunophenotyping panel. Whole blood samples were processed (fixed, barcoded, and stained) as described in the protocol. Gaussian parameter cleanup and doublet exclusion (refer to Figure 2 for details) were performed before T-cell delineation. (A) Bivariate plots demonstrate the hierarchical gating strategy for T-cell subsets in a representative healthy donor. T cells were first identified as CD3^+^CD56-. These were then subdivided into helper T cells (CD4^+^) and cytotoxic T cells (CD8^+^). Helper T cells were further classified into two categories: activated regulatory T cells (activated Tregs; CD127-CD25^+^) and CD4^+^ T effector (CD4^+^ Teff) cells. Both CD8^+^ and CD4^+^ T-effector cells were categorized based on their expression of CD27 and CD45RA into effector memory (TEM; CD27-CD45RA-), central memory (TCM; CD27^+^CD45RA-), naive (Tnaive; CD27^+^CD45RA^+^), or terminally differentiated (TEMRA; CD27-CD45RA^+^) subsets. (B) Bar graph depicting the relative frequency (%) of T-cell subsets in healthy donor whole blood (n = 4). Bars represent the average relative frequency of each subset, and error bars indicate the standard deviation (SD) across three technical replicates of the four healthy donors.

## Data analysis

After acquiring samples, the data were normalized using CyTOF Software (Standard BioTools, Version 7.1), which allows the normalization of CyTOF data to a global standard known as a bead passport. This standard has been determined for each EQ4 bead lot and consists of the mean dual instrument (Di) counts for all masses of the specified bead lot, as established during the manufacturing process. The EQ4 element calibration beads contain isotopes of cerium (140/142Ce), europium (151/153Eu), holmium (165Ho), and lutetium (175/176Lu). Normalization factors are determined using critical isotopes (140Ce, 151/153Eu, 165Ho, and 175Lu) and mass channels between the encoded isotopes. Alternative isotope normalization factors are obtained through linear interpolation or extrapolation. Subsequently, all mass channel event values are multiplied by these normalization factors to produce normalized values, which are then saved to a normalized file [7].

**Figure 5.**
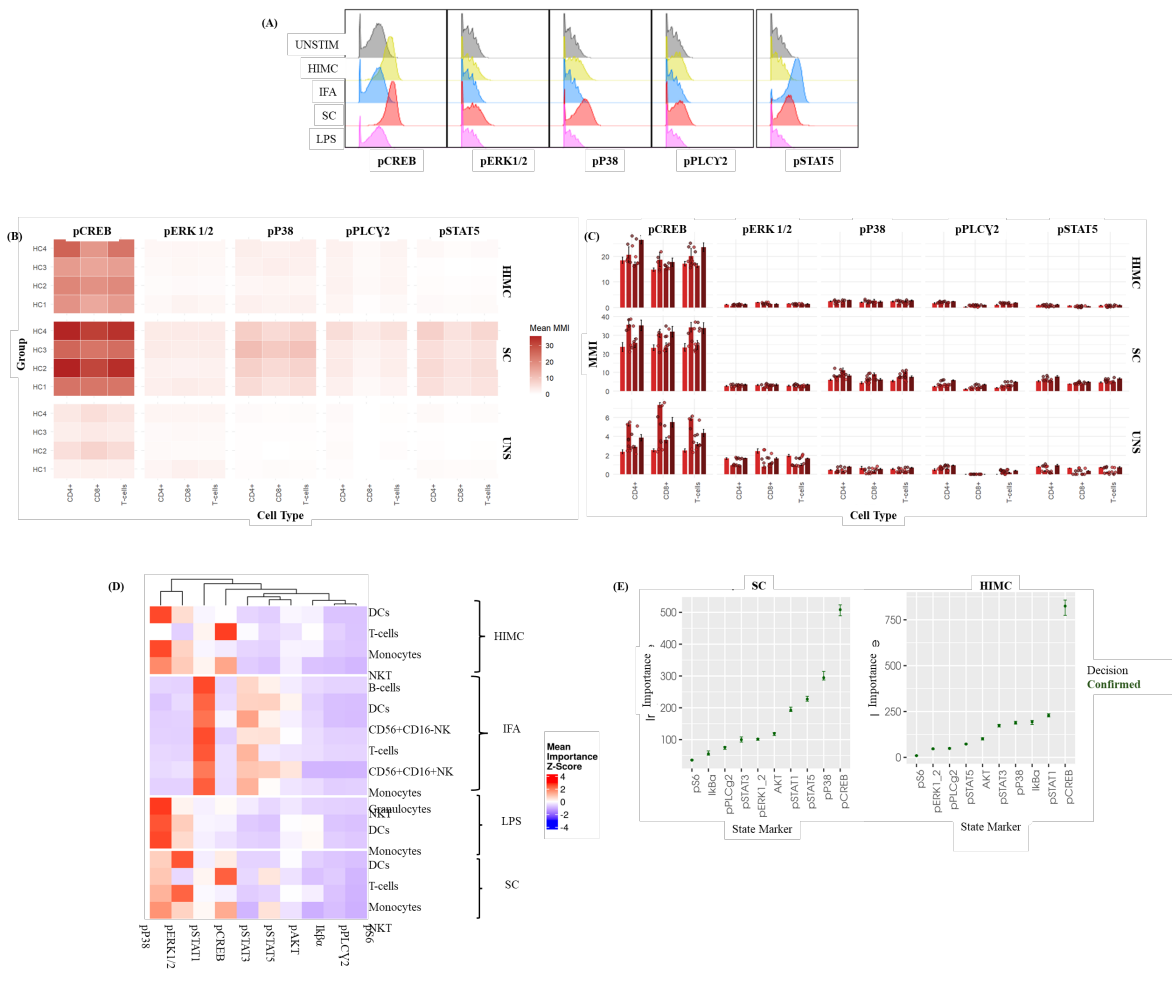
Intracellular phospho-profiling of T-cells in healthy whole blood in response to ex vivo stimulation. This figure presents the results of intracellular phospho-protein profiling in T cells from healthy whole blood, following ex vivo stimulation with various activators. Whole blood samples were processed according to a detailed protocol, which included fixation, barcoding, and staining with a comprehensive panel of surface and intracellular markers to assess phosphorylation status. (A) Representative histograms illustrating the distribution of specific phospho-marker expression within the total T-cell population after stimulation. These histograms highlight the heterogeneity in phosphorylation responses, exemplified by the p38 response to the Human Immune Monitoring Center (HIMC) protocol and pSTAT5 from the StemCell (SC) activator cocktail. Data is from a single, representative healthy donor. (B) Heatmaps displaying differences in median metal intensity (MMI) for various phospho-proteins at baseline (UNSTIM) and after T-cell stimulation with the StemCell (SC) activator cocktail and the HIMC protocol. Each row represents a healthy donor stimulation combination, and each column represents a phospho-protein. The heatmaps illustrate overall differences in phosphorylation across CREB, ERK 1/2, P38, PLCγ2, and STAT5. (C) Bar graphs representing the median metal intensities (MMIs) of specific phospho-proteins within defined T-cell subsets after stimulation with the StemCell (SC) activator cocktail and the HIMC protocol. Bars depict the average MMI, and error bars indicate the standard deviation (SD) across four healthy donors (biological replicates), each with three technical replicates. (D) Representative HDStIM heatmap comparing the mean importance scores for each significant stimulation population combination (SPC) after 15 min of ex vivo stimulation with IFNα, HIMC protocol, SC activator cocktail, or LPS. Each row in the heatmap corresponds to a unique population–stimulation combination, and each column corresponds to a specific phospho-protein. Heatmap coloring reflects z-scores, indicating the relative importance of each phospho-protein in defining a particular stimulation-response phenotype within that population. Data are derived from the average responses of four healthy donors (biological replicates) with three technical replicates as determined by HDStIM analysis. (E) T-cell marker rankings by importance for the SC and HIMC stimulation conditions in healthy whole blood. This panel presents the relative contribution of individual phospho-proteins to the overall stimulation response signatures, as determined by HDStIM analysis, providing insight into the most impacted signaling pathways under each condition. The data represents average responses from four healthy donors (biological replicates) with three technical replicates.

Analysis of raw FCS files was performed using FlowJo software to clean the data using Gaussian parameters (refer to Figure 2) and assess all subsets based on the dual-count signal in each mass channel. The percentage of each cell type is determined (refer to Figure 3 for quantification of all major immune subsets or Figure 4 for all T-cell subsets) and reported as the frequency of total (FT%) or absolute count, which is calculated by multiplying the frequency of total for each cell type by the total cells run for that barcoded sample. Median metal intensity (MMI) values for each intracellular epitope allow the quantification of phosphorylation for each protein in response to stimulation. All negative raw MMI values were corrected to zero, and all values equivalent to zero in unstimulated samples were corrected to one (the baseline). To manually calculate fold change for each intracellular cell-state marker, we used the formula MMI_stim_/MMI_unstim_ for each donor. GraphPad Prism was used to plot the resulting data, demonstrating differences in fold change for each cell population–stimulation combination. Alternatively, gated FCS files for each cell population–stimulation combination were transferred for bioinformatic analysis using the HDStIM pipeline (refer to Figure 5D, E and Figure S1). By comparing phosphorylation levels between unstimulated and stimulated cells, we can gain insight into the status of the immune system [1].

An internally developed R script was employed to convert the FlowJo export tables into graphics [8]. Written for R 4.3 in RStudio [9], the script (i) ingests the worksheets generated at the gating step, (ii) harmonizes donor, replicate, and stimulation metadata, and (iii) reshapes the frequency and median-intensity matrices into a tidy long format with readxl, dplyr, tidyr, and stringr [10–12]. Plotting is performed with ggplot2 [13], while color management relies on RColorBrewer, viridis, and scales [14–16] to ensure palette consistency across figures. The same script then (a) summarizes relative immune-subset frequencies (mean ± SD or 0%–100% normalized compositions) (refer to Figures 3 and 4), (b) visualizes baseline median metal intensities (refer to Figure 5B, C for UNSTIM), and (c) displays stimulation-induced fold-changes (refer to Figure 5B, C for SC and HIMC), and exports all figures automatically in both raster (JPEG) and vector (PDF) formats.

**HDStIM**

Flow cytometry standard (FCS) files were processed using the flowCore R package, with raw expression data extracted and marker channels mapped according to experimental panel configuration. An inverse hyperbolic sine (arcsinh) transformation with a cofactor of 5 was applied to all protein expression markers to normalize expression distributions and stabilize variance. High-dimensional stimulation immune mapping (HDStIM) was performed to identify stimulus-responsive cell populations by applying k-means clustering (k = 2) to combined stimulated and unstimulated cells, using all state markers for each cell population–stimulation combination. Fisher's exact test compared cluster membership distributions between conditions, with combinations showing p < 0.05 considered to contain stimulus-responsive populations. The Boruta algorithm identified markers distinguishing between responding and non-responding cells by comparing feature importance scores with those from randomly permuted controls. P-values were adjusted using Benjamini–Hochberg false discovery rate correction for multiple comparisons.

Quality control included three diagnostic visualizations for each responding population: 1) stacked bar charts of Fisher's exact test results, 2) UMAP plots showing cell separation based on functional marker expression, and 3) expression distribution plots comparing unstimulated, stimulated, and responding populations. Marker importance scores were visualized as heatmaps using both min-max normalization (0–1 scale) and z-score standardization. All analyses were implemented in R (version 4.2.0) using flowCore (v2.10.0) [17], HDStIM (v0.1.0), Tidyverse (v2.0.0) [18], ComplexHeatmap (v2.14.0) [19,20], Arrow (v14.0.0) [21], and Boruta (v8.0.0) [22] packages.

**Quantification and statistical analysis**

Manual gating is employed to determine the frequency of total (%) immune cell populations (refer to Figure 3). Differentially abundant cell populations are identified using univariate tests, such as the t-test, Mann–Whitney test, or one-way ANOVA with a multiple comparison test. Additionally, the association between cell frequencies and different outcomes can be examined using linear or logistic regression.

## Validation of protocol

This protocol was optimized using Jurkat cl E6-1 immortalized lymphoblastic leukemia cells and a smaller surface panel of antibodies, including CD45, CD3, and CD4, and the full panel of intracellular antibodies as described in Table 5. The HIMC protocol was used to establish the phospho-response to stimulation with anti-CD3 (refer to Figure S2). Since endogenous T-cell activation requires both TCR engagement and co-stimulation, the SC activator cocktail, which is a proprietary reagent composed of both anti-CD3 and anti-CD28, was titrated and tested for its ability to recapitulate phospho-responses achieved with the HIMC protocol (refer to Figure S3). Results confirmed the use of the SC activator cocktail at a mid-dose of 50 µL/well and recapitulated the manufacturer’s suggestions. Using both T-cell stimuli, the kinetics of phosphorylation for the proteins involved in T-cell activation were assessed (Figure S4), and a 15-min stimulation length with the SC activator cocktail and the HIMC protocol was determined optimal for all further studies. Next, the optimized conditions were validated in healthy donor whole blood (n = 3) (refer to Table S1 and S2) using the surface and intracellular master mix panels described in Tables 4 and 5. An unstimulated control was included for each donor to normalize and calculate the fold change in median metal intensities for all intracellular cell state markers. Unstimulated samples were also used to establish the baseline phosphorylation status of the cell state proteins before stimulation (refer to Figure 5B and C for UNSTIM). IFNα and LPS stimuli were used as assay controls to confirm the success of the experiment (refer to Figure S5). Stimulation with IFNα elicits the phosphorylation of STAT1, STAT3, and STAT5 in the primary immune cell subsets, including T cells, B cells, natural killer (NK) cells, and monocytes (refer to Figure S6). In myeloid subsets, LPS stimulation triggers the phosphorylation of p38, ERK1/2, and the degradation of IκBα (refer to Figure S5). Data obtained using the described protocol were compared with previously generated data in our lab [6] and the literature to confirm phosphorylation of proteins following T-cell stimulation. Representative data for healthy whole blood is available upon request.

## General notes and troubleshooting

**General notes**

A. Sample collection and preparation

1. **Collection:** Use only **freshly drawn whole blood (within ~2 h of draw) collected in sodium heparin tubes (green tops)**. Do not use EDTA or citrate collection tubes. As an anticoagulant, both EDTA and citrate can modulate calcium signaling, which may interfere with downstream analyses.

2. Resting: To ensure uniform cellular responses to stimulation, **rest whole blood for a minimum of 30 min and a maximum of 1 h at 37 °C** before proceeding with the 15-min stimulation.

3. Volume: As specified by SmartTube Inc., **a whole blood volume of 200 μL from a healthy donor** is sufficient to delineate major immune cell populations. Conversely, a larger processing volume may be required if donors are leukopenic or the assay involves the investigation of rare cell populations. In this instance, we recommend validating the thaw/lyse protocol.

B. Stimuli preparation and handling

1. Stimuli banking: We recommend **preparing and aliquoting stimuli into matrix color-coded tubes** for banking and standardization and to limit batch variability.

2. Stimuli storage: **Stimuli can be prepared fresh on the day of the assay or in advance and stored at -80 °C**. To maintain reagent integrity, avoid freezing and thawing stimuli more than twice.

3. Stimuli concentration: Prepare **stimuli at a 5× concentration** in cRPMI (stimulation media) and aliquot **50 μL into matrix tubes**.

4. Stimulation conditions: Since phosphorylation events occurring within a cell are dynamic, the **stimulation temperature and time should be strictly adhered to for assay reproducibility**. Alternative stimulation conditions should be tested and validated prior to execution.

C. Lysis and sample containers

1. Red blood cell lysis: **Add thaw/lyse buffer to whole blood at a 4:1 ratio**. Typically, red blood cell lysis is achieved through two lysis cycles. If residual red blood cells are observed, **a third lysis cycle may be necessary**; however, this can impact the signal intensities of barcodes and all staining markers and potentially increase the acquisition time on the CyTOF.

2. Sample processing: **Simultaneous processing of multiple whole blood samples benefits from the use of a 2 mL deep-well plate**. For fewer samples, polystyrene 5 mL round-bottom tubes can be used as an alternative.

3. Artifacts from whole blood lysis: Several artifacts with the potential to up- or downregulate phosphorylation may interfere with accurate measurement of phosphorylation. Hence, the most important factor to mitigate these artifacts is **performing immediate and effective fixation prior to whole blood lysis**.

D. CyTOF acquisition and instrument settings

1. Sample resuspension and stability: **Immediately before running** a barcoded set on the Helios, **resuspend that sample in CAS^+^ solution containing a 1:10 dilution of EQ4 beads. Prolonged storage of cells in CAS^+^ can lead to significant cell loss**.

2. Sample filtering: To **prevent clogging and cell clumping** during acquisition, **filter samples through a 5 mL Falcon tube with a straining cap immediately before loading onto the instrument**.

3. Acquisition concentration: Whole blood samples can be **run at a concentration of 1.3 × 10^6^ cells/mL** to achieve an **optimal acquisition rate of 350 events/s (± 50 events/s)**.

4. Ionization and cell loss: Approximately **40%–60% of cells are lost due to the ionization process** and other technical factors on the Helios CyTOF. Therefore, **only 40%–60% of the cells loaded onto the machine will be detected**. Take this into account when planning experiments where rare cell populations will be analyzed.

5. Target event count: Aim to **acquire approximately 100,000–150,000 events per barcoded sample** for robust downstream analysis.

6. **Instrument preparation:** Before running samples, ensure the use of a wide-bore injector and precondition the Helios instrument with CAS^+^.

7. Between-sample washes: **Run ultra-pure water for 5 min between each barcoded sample** set to remove adherent lanthanides from the sample capillary. **Monitor the event rate in preview mode until it falls below one event per second** to confirm capillary cleanliness.

8. Length of acquisition: For longer runs, such as those taking 8–12 h, **tuning should be repeated every 4–6 h** to ensure consistency. This can add about 30 min of setup per tuning session, including wash cycles.

E. Limitations

1. For HDStIM analysis, we employ nominal p-values without multiple testing corrections, which are typically used for exploratory analysis to demonstrate the tool's value rather than to define differential immune phenotypes [6] definitively. Alternative data analysis packages, such as CATALYST, can be used for more definitive assessments of immune phenotypes.

2. The Helios operates at a maximum acquisition rate of 350 events per second, with a variation of ±50 events per second. Running at higher speeds may introduce clogs and skew results.

3. In a single day, one individual can comfortably run a maximum of four barcoded sets, totaling 80 samples.

4. At least 1 million cells should be run per barcoded set on the Helios. Take into consideration that up to 60% of the sample will be lost due to ionization.

5. As the time run progresses, instrument drift may occur, introducing variability in the data.

6. After a long day of acquisition, the Helios requires deep cleaning to reduce instrument drift and variability, to maintain sensitivity and quality of signal, and to prevent clogging.

**Troubleshooting**

**Problem 1:** Unexpected heavy metal contamination in samples and acquired data.

**Possible cause:** Several commonly used laboratory detergents contain heavy metals (i.e., barium).

**Solution:** Avoid using reusable laboratory consumables (e.g., glass beakers and pipettes) that have been cleaned with detergent to prepare staining buffers or master mixes. Utilize metal-free reagents and plastics where possible.

**Problem 2:** Insufficient cell yield or loss.

**Possible cause:** Failure to meet the minimum cell requirement for barcoded sample sets (i.e., 1.5–2 × 10^6^ cells). Significant cell loss, particularly after methanol permeabilization.

**Solution:** Implement barcoding and pooling cells early in the protocol to mitigate overall cell loss. Additionally, increase the centrifugation speed/duration after methanol permeabilization (i.e., 1,000–1,200× *g* for 10 min) to improve cell pelleting and yield.

**Problem 3:** Poor phospho-marker signal intensity.

**Possible cause:** Incomplete cell fixation, insufficient permeabilization, incorrect antibody titrations, or expired/degraded reagents.

**Solution:** Ensure the fixation protocol is optimized for whole blood, always use 100% ice-cold methanol for permeabilization, titrate phospho-antibodies on fresh samples to confirm optimal concentration before running the complete panel, and confirm antibody stability and expiration dates.

**Problem 4:** High background and/or nonspecific staining.

**Possible cause:** Antibodies are over-concentrated, inadequate washing, potential protein degradation, or eosinophils may show nonspecific binding of metal-labeled antibodies due to a charge-based interaction between antibodies and the cationic proteins located within the granules of eosinophils.

**Solution:** Titrate antibodies using the appropriate cells/controls, increase the number of washes, validate the antibody lot consistency, and include the appropriate negative controls (unstained samples, isotype controls) to identify nonspecific binding. For eosinophils, implement a blocking step with an anionic protein like heparin.

**Problem 5:** Cell loss following methanol permeabilization.

**Possible cause:** Insufficient centrifugation speed/duration.

**Solution:** Increase centrifugation speed/duration after methanol permeabilization (i.e., 1,000–1,200× *g* for 10 min).

**Problem 6:** Inconsistent stimulation response across donors.

**Possible cause:** Intrinsic biological donor variability or inconsistent stimulation conditions; the stress of physical manipulation and sample processing may cause cell activation. Delay in or prolonged lysis may permit the occurrence of nonspecific signaling events, creating artificial phosphorylation signals.

**Solution:** Standardize timing (kinetics), temperature, and stimulation concentrations; include known controls like IFNa or LPS to confirm assay success or optimize lysis timing.

**Problem 7:** Poor reproducibility between runs or experiments.

**Possible cause:** Batch variability, stemming from differences in sample processing or reagent lots.

**Solution:** Implement barcoding to limit batch variability. For batch normalization, run all samples on the same day using the same antibody lots for staining mixes.

**Problem 8:** Cell clumping/clogging during Helios acquisition.

**Possible cause:** Insufficient washing, residual red blood cell debris, or prolonged incubation in CAS^+^.

**Solution:** Filter all samples immediately before acquisition. Limit the time in CAS^+^ by processing one barcoded sample at a time for acquisition on the Helios. Ensure that RBCs are lysed prior to barcoding and staining.

**Problem 9:** Low event rate when acquiring samples on the Helios.

**Possible cause:** Insufficient cell concentration, partial clogs in the instrument, or debris in samples.

**Solution:** Do not run samples that are too diluted; adjust to a smaller volume if necessary. Routinely implement checking the nebulizer for clogs. After a lengthy acquisition, thoroughly clean the nebulizer by implementing 1 N NaOH prior to water and Contrad. If an unclogged nebulizer is available, switch to it to complete the run. Also, ensure that samples are filtered through a 5 mL Falcon strainer cap tube immediately before acquisition to remove debris.

**Problem 10:** Altered phosphorylation status, increased nonspecific antibody binding, loss of leukocytes.

**Possible cause:** Artifacts resulting from insufficient whole blood lysis. Incomplete RBC lysis may promote leukocyte clumping, including T cells, with residual RBCs or debris. This leads to a loss of cells during processing, poor sample collection, and inaccurate downstream analysis, especially for rare cell populations.

**Solution:** Implement fixation protocols that contain formaldehyde. Always perform sample fixation prior to lysis to prevent enzymatic activity during the lysis step. Ensure the use of optimized lysis reagents and protocols. Consider implementing blocking to prevent nonspecific binding. For example, heparin (used in the green-top whole blood collection tubes) aids in preventing nonspecific staining in cells, including eosinophils.

## Supplementary information

The following supporting information can be downloaded here:

1. Table S1. NIH Clinical Center’s Blood Bank Donor Race Key.

2. Table S2. Healthy whole blood donor information.

3. Figure S1. Multi-parameter mapping of T-cell signaling states using HDStIM.

4. Figure S2. Phosphorylation of CREB and S6 indicates T-cell activation.

5. Figure S3. StemCell activator cocktail reveals similar phosphorylation patterns to those achieved with the HIMC protocol in PBMCs.

6. Figure S4. Kinetics of phosphorylation in response to T-cell stimulation reveal temporal differences in the phosphorylation of CREB, S6, PLCγ2, ERK1/2, and STAT5.

7. Figure S5. Marker-by-marker analysis of phosphorylation responses to IFNα and LPS in healthy whole blood.

8. Figure S6. Phospho-responses of healthy donor whole blood stimulated with IFNα, LPS, HIMC, and SC activator cocktail.

## References

[1] Fernandez R. and Maecker H. (2015). Cytokine-Stimulated Phosphoflow of Whole Blood Using CyTOF Mass Cytometry. Bio Protoc. 5(11): e1495. 10.21769/BioProtoc.1495 PMC4847742 27135045

[2] Ramos I., Han K. L., Farmer R. and Douagi I. (2023). Integrating spectral and mass cytometry for a higher resolution single-cell snapshot of the immune system. Presented at the ISAC CYTO Conference, May 6-10, 2023, Montreal, Canada. https://cdn.ymaws.com/isac-net.org/resource/resmgr/docs/cyto_programs/cyto23_program_v6_-_final.pdf.

[3] Castro-Sanchez P., Teagle A. R., Prade S. and Zamoyska R. (2020). Modulation of TCR Signaling by Tyrosine Phosphatases: From Autoimmunity to Immunotherapy. Front Cell Dev Biol. 8: 608747 10.3389/fcell.2020 .608747 33425916 PMC7793860

[4] Bandura D. R., Baranov V. I., Ornatsky O. I., Antonov A., Kinach R., Lou X., Pavlov S., Vorobiev S., Dick J. E. and Tanner S. D. (2009). Mass Cytometry: Technique for Real Time Single Cell Multitarget Immunoassay Based on Inductively Coupled Plasma Time-of-Flight Mass Spectrometry. Anal Chem. 81(16): 6813 6822 6822. 10.1021/ac901049w 19601617

[5] Haas A., Weckbecker G. and Welzenbach K. (2008). Intracellular Phospho‐Flow cytometry reveals novel insights into TCR proximal signaling events. A comparison with Western blot. Cytometry Part A. 73A(9): 799–807. 10.1002/cyto.a .20598 18548611

[6] Farmer R., Apps R., Quiel J., Sellers B. A., Cheung F., Chen J., Mukherjee A., McGuire P. J. and Tsang J. S. (2022). Multiparameter stimulation mapping of signaling states in single pediatric immune cells reveals heightened tonic activation during puberty. bioRxiv. https://doi.org/10.1101/2022.1114.516371.

[7] Bagwell C. B., Inokuma M., Hunsberger B., Herbert D., Bray C., Hill B., Stelzer G., Li S., Kollipara A., Ornatsky O., .(2019). Automated Data Cleanup for Mass Cytometry. Cytometry Part A. 97(2): 184 198 198. 10.1002/cyto.a .23926 31737997

[8] Bryan J. and Wickham H. (2023). readxl: Read Excel Files. R package version 1.4.3.

[9] R Core Team(2025). R: A Language and Environment for Statistical Computing. R Foundation for Statistical Computing, Vienna, Austria.

[10] Wickham, H. et al.(2023). dplyr: A Grammar of Data Manipulation. R package version 1.2.4.

[11] Wickham H. and Girlich M. (2024). tidyr: Tidy Messy Data. R package version 1.3.0.

[12] Wickham H. (2023). stringr: Simple, Consistent Wrappers for Strings. R package version 1.5.1.

[13] Wickham H. (2016). ggplot2: Elegant Graphics for Data Analysis. Springer-Verlag, New York.

[14] Neuwirth E. (2014). RColorBrewer: ColourBrewer Palettes. R package version 1.1-3.

[15] Garnier S. (2024). viridis: Colourblind-Friendly Colour Maps. R package version 0.6.5.

[16] Wickham H. and Seidel D. (2022). scales: Scale Functions for Visualization. R package version 1.3.0.

[17] Ellis B, Haaland P, Hahne F, Le Meur N, Gopalakrishnan N, Spidlen J, Jiang M, Finak G (2025). flowCore: flowCore: Basic structures for flow cytometry data. doi: 10.18129/B9.bioc.flowCore , R package version 2.10.0, https://bioconductor.org/packages/flowCore

[18] Wickham H., Averick M., Bryan J., Chang W., McGowan L., François R., Grolemund G., Hayes A., Henry L., Hester J., .(2019). Welcome to the Tidyverse. J Open Source Softw. 4(43): 1686 10.21105/joss.01686

[19] Gu Z., Eils R. and Schlesner M. (2016). Complex heatmaps reveal patterns and correlations in multidimensional genomic data. Bioinformatics. 32(18): 2847 2849 2849. 10.1093/bioinformatics/btw313 27207943

[20] Gu Z. (2022). Complex heatmap visualization. iMeta. 1(3): e43. https://doi.org/10.1002/imt2.43 PMC10989952 38868715

[21] Richardson N, Cook I, Crane N, Dunnington D, François R, Keane J, Moldovan-Grünfeld D, Ooms J, Wujciak-Jens J, Arrow Apache (2025). arrow: Integration to'Apache''Arrow'. R package version 14.0.0, https://github.com/apache/arrow

[22] Kursa MB, Rudnicki WR (2010). Feature Selection with the Boruta Package. J Stat Softw. 36(11): 1 13 13. 10.18637/jss.v036.i11

[23] Marsh‐Wakefield F. M., Mitchell A. J., Norton S. E., Ashhurst T. M., Leman J. K., Roberts J. M., Harte J. E., McGuire H. M. and Kemp R. A. (2021). Making the most of high‐dimensional cytometry data. Immunol Cell Biol. 99(7): 680 696 696. 10.1111/imcb.12456 33797774 PMC8453896

[24] Rybakowska P., Van Gassen S., Martorell Marugán J., Quintelier K., Saeys Y., Alarcón-Riquelme M. E. and Marañón C. (2022). Protocol for large scale whole blood immune monitoring by mass cytometry and Cyto Quality Pipeline. STAR Protoc. 3(4): 101697 10.1016/j.xpro .2022.101697 36353363 PMC9637821

